# Burden of Eating Disorders in China, 1990-2019: An Updated Systematic Analysis of the Global Burden of Disease Study 2019

**DOI:** 10.3389/fpsyt.2021.632418

**Published:** 2021-05-21

**Authors:** Zhitao Li, Lili Wang, Haixia Guan, Cheng Han, Peng Cui, Aihua Liu, Yongze Li

**Affiliations:** ^1^Department of Psychiatry and Psychological Clinic, Affiliated Quanzhou First Hospital, Fujian Medical University, Quanzhou, China; ^2^Department of Psychiatry, The First Hospital of China Medical University, Shenyang, China; ^3^Department of Psychiatry, Quanzhou Third Hospital, Quanzhou, China; ^4^Department of Endocrinology, Guangdong Provincial People's Hospital, Guangdong Academy of Medical Sciences, Guangzhou, China; ^5^Department of Clinical Nutrition and Metabolism, Affiliated Zhongshan Hospital of Dalian University, Dalian, China; ^6^Department of Interventional Radiology, Chengdu Municipal Third People's Hospital, Chengdu, China; ^7^Department of Endocrinology and Metabolism, Peking University Third Hospital, Beijing, China; ^8^Department of Endocrinology and Metabolism and the Institute of Endocrinology, The First Hospital of China Medical University, Shenyang, China

**Keywords:** eating disorder, anorexia nervosa, bulimia nervosa, incidence, prevalence, China

## Abstract

**Background:** Eating disorders, including anorexia nervosa (AN) and bulimia nervosa (BN), are complex mental disorders. A better understanding of the burden of eating disorders is essential for improving their management. Information about the burden of eating disorders at the national level in China remains unclear.

**Methods:** This is a systematic analysis of the Global Burden of Disease Study (GBD) 2019. The sex- and age-specific prevalence, incidence, and disability-adjusted life years (DALYs) of eating disorders in China were estimated by systematically reviewing all available epidemiological data and inputting these data into a Bayesian meta-regression tool (DisMod-MR 2.0). Trends in the age-standardized prevalence, incidence, and DALYs due to AN and BN were assessed from 1990 to 2019.

**Results:** The age-standardized incidence rate (ASIR), prevalence rate (ASPR), and DALY rate per 100,000 population were estimated to be 13.22 (95% UI, 9.35–18.23), 38.08 (95% UI: 26.37–55.73), and 8.38 (95% UI, 4.87–13.35) for AN and 130.05 (95% UI, 84.02–187.13), 75.21 (95% UI, 48.52–105.97), and 16.16 (95% UI, 9.23–25.40) for BN, respectively, in 2019. The prevalence, incidence, and DALY rate of AN peaked at 15–19 years old. The prevalence and DALY rate of BN peaked at 30–34 years old. Females had a higher burden of AN and a lower burden of BN than males. The ASIR, ASPR, and DALY significantly increased by 1.3% (95% CI: 1.3–1.4%), 1.6% (95% CI, 1.5–1.6%), and 1.6% (95% CI, 1.5–1.7%) for AN and 1.4% (95% CI: 1.4–1.4%), 2.0% (95% CI, 2.0–2.1%), and 2.0% (95% CI, 2.0–2.1%) for BN, respectively, from 1990 to 2019 in China. In addition, the increments in all the age-standardized measures of BN were higher in males than in females.

**Conclusions:** The burden of eating disorders in China showed unexpected patterns that varied by sex and age, with increasing trends of AN and BN from 1990 to 2019. More attention should be given to improving the burden of BN in males in China.

## Introduction

Eating disorders, including anorexia nervosa (AN) and bulimia nervosa (BN), are serious and complex mental disorders. AN is a severe psychiatric disorder that is characterized by starvation and malnutrition, a high incidence of coexisting psychiatric conditions, treatment resistance, and a substantial risk of death from medical complications and suicide (1). Bulimia nervosa is characterized by recurrent episodes of binge eating and compensatory behaviors to prevent weight gain (2). The underlying mechanisms of eating disorders might be associated with interactions between genetic and environmental factors at a crucial period in development (3). Genome-wide association studies involving large international cooperative efforts have identified eight loci associated with AN (4, 5). Longitudinal studies suggest that environmental influences in the perinatal period are of relevance for AN, whereas problems in early childhood are associated with binge spectrum disorders (6, 7). Numerous studies have shown that a substantial disease burden, decreased quality of life, and increased risk of depression, substance abuse, and suicide are associated with eating disorders (8–13).

In 2013, ~1.9 million (1.2–2.8 million) disability-adjusted life years (DALYs) were associated with eating disorders worldwide (14). It was estimated that eating disorders accounted for 0.1% of total DALYs worldwide and 24.6 (15.8–36.5) per 100,000 age-standardized DALYs (14). The DALY rates of AN and BN are 1.9 (1.2–2.8) and 6.9 (4.1–11.0) per 100,000 in men and 11.0 (7.4–15.7) and 30.2 (18.8–46.1) per 100,000 in women, respectively (14). The DALY rate for eating disorders did not change significantly from 1990 (24.8 per 100,000) to 2013 (14).

The prevalence of eating disorders has great regional variations, and the difference between the proportions of DALYs attributable to eating disorders could be a multiple of 40 times among countries (15). Previous studies have shown that the age-standardized DALY rates for AN and BN in high-income countries are far higher than those in low- and middle-income countries (14, 15). The incidence of AN in Europe has stabilized since the 1970s (16, 17). The overall incidence of AN was stable in the Netherlands, but the incidence in females aged 15–19 years was significantly higher in the 1990s than in the 1980s (18). The incidence of BN has decreased after a sharp rise in the UK (16). It is indeed that the high burden of eating disorders remains in the high-income western regions, but an increasing trend was observed worldwide, particularly in Asian regions that were highly populous (19). Historical evidence regarding the epidemic of eating disorders is commonly studied in Western countries; however, studies in China are mainly limited to Hong Kong, which is a high-income region (20, 21). Several studies have investigated the prevalence of eating disorders in mainland China, with adolescents or young adults as the target populations for research and prevention (22–24).

A recent study explored the secular trends in the incidence of eating disorders in China from 1990 to 2017 (25). Although the incidence metric is essential for prevention strategies, they provide one sole indicator of assessment when reviewed individually. The prevalence of eating disorders is also important to guide the allocation of resources for management. DALYs were used to measure health loss due to the burden of both fatal and non-fatal diseases. An understanding of the trends over time of these metrics will guide future public health policy assessments and optimal resource allocation. Moreover, the sociodemographic index (SDI) is a comprehensive measurement of education level, income per capita, and fertility rate. No study has examined the relationship between the burden of eating disorders and sociodemographic index in China. In addition, the 2019 Global Burden of Disease (GBD) Study Group published updated GBD estimations in October 2020, which provides a standardized methodology to estimate the disease burden of eating disorders by sex, age, year, and location (26). In GBD 2019, eating disorders were only divided into AN and BN, which contributed to a large proportion of related cases. Therefore, we focused on AN and BN to describe eating disorders in the current study. As the first step to elucidating eating disorder status in China, we used the GBD 2019 to describe the age-standardized incidence, prevalence, and DALYs of eating disorders in 2019. We also aimed to examine the temporal trends of eating disorders from 1990 to 2019 in China by using join point regression analysis. We further compared the trends of the annual SDI and these metrics of eating disorders in China.

## Materials and Methods

### Data Sources

The GBD 2019, which covered 204 countries and regions from 1990 to 2019, provided a comprehensive assessment of health loss for 369 diseases and injuries (26). Details of the methodology used in GBD 2019 are described on the official website (http://ghdx.healthdata.org/gbd-results-tool). The GBD 2019 used systematic reviews, survey data, hospital administrative data, disease registries, inpatient and outpatient data, claims, and case notifications as data sources to estimate disease incidence. The targeted systematic review of the eating disorders dataset utilized the China National Knowledge Infrastructure database to find studies. For those countries where raw data were not available, DisMod-MR 2.0 produces an imputed estimate for each epidemiological parameter based on available data from surrounding countries. The raw epidemiological data used to inform the prevalence estimates were required to meet strict inclusion criteria, including being representative of the general population and therefore not including any treatment or clinical samples. Population estimates and confidence intervals were produced via Bayesian statistical methods. The datasets generated for this study can be found in the GBD at http://ghdx.healthdata.org/gbd-results-tool. We abstracted the data and further conducted systematic analysis for this topic.

### Definition of Eating Disorders

Eating disorders are divided into two major categories in GBD studies: AN and BN. Included in GBD were cases meeting diagnostic criteria according to the Diagnostic and Statistical Manual of Mental Disorders (DSM) or ICD. Different versions of the DSM (DSM-III, DSM-III-R, DSM-IV, DSM-IV-TR, and DSM-5) and ICD (ICD-9 and ICD-10) were accepted (27–29).

### Estimates of Disease Burden

The general methodology of GBD 2019 has been introduced elsewhere (26). In brief, the methodological framework began with estimating mortality. By using the cause of death ensemble model, various data from different sources were obtained to generate estimations of mortality from specific causes. In this process, the incidence and mortality data from different sources were analyzed with a linear-step mixed-effects model and sociodemographic index (a comprehensive indicator of income, education level, and fertility) to generate crude mortality-to-incidence ratios (MIRs) as the predictive covariates, and then general spatiotemporal Gaussian process regression was performed to obtain the final MIR. The estimated incidence was obtained by dividing the final mortality estimate by the MIR and using the MIR and survival estimates to calculate the 10-year prevalence estimate. Years of life lost (YLLs) were estimated by multiplying the estimated number of deaths by the age of the patients and the standard life expectancy of the corresponding age. Years lived with disability (YLDs) were estimated by multiplying the prevalence and a distinct disability weight in the Bayesian regression model. DALYs were calculated by summing the YLLs and YLDs. The age-standardized rate was calculated on the basis of the following formula:

$$\begin{array}{l} \text{Age}-\text{standardized rate}=\frac{\sum_{i=1}^{\text{A}}{{\text{a}}_{\text{i}}w}_{i}}{\sum_{i=1}^{\text{A}}w_{i}}\times 100,000 \end{array}$$

The age-standardized rate per 100,000 population is equal to the sum of the products of age-specific rates (w_i_, where *i* denotes the *i*th age class) and number of cases (or weight; w_i_) in the same age subgroup *i* of the selected reference standard population and then divided by the sum of the standard population weights (26). Age-standardized rates were calculated considering the GBD world population.

### Statistics

The burden of eating disorders is represented by age-standardized prevalence rates (ASPRs), age-standardized incidence rates (ASIRs), age-standardized mortality rates (ASMRs) and age-standardized DALYs. Joinpoint regression analysis was used to assess trends in the disease burden of eating disorders. Joint Command Line Version 4.5.0.1 joinpoint software was provided by the United States National Cancer Institute Surveillance Research Program. This software tracks trends in data over time (for the present analysis, ASIR, ASPR, and DALYs) and then fits the simplest model possible to the data by connecting several different line segments on a logarithmic scale. These segments are known as “Joinpoints,” with the simplest model (i.e., 0 Joinpoints) being a straight line. As more joinpoints are added, each is tested for significance using a Monte Carlo permutation method. The logarithmic transformation of the rates was conducted, and the standard errors were calculated based on binomial approximation (30). The estimated annual percent change (APC) and 95% confidence interval (CI) were calculated for each metric trend by fitting a regression line to the natural logarithm of the observed rates using the calendar year as a regression variable (31). The average APC (AAPC) was also estimated by assuming one segment for the full range of our study periods. The *Z* test was used to assess whether the APCs were significantly different from zero. When describing trends, the terms increase or decrease are used when the slope of the trend is statistically significant. The 95% uncertainty interval (UI) for each quantity was calculated in our study. The sex-specific crude rates and 95% UIs were used to compare the burden of eating disorders between age groups: 5–9, 10–14, 15–19, 20–24, 25–29, 30–34, 35–39, 40–44, and 45–49. Significance in all the analyses was assessed at the 0.05 level, and all hypothesis tests were two-sided.

## Results

### Eating Disorder Burden in China, 2019

The ASIR, ASPR, and age-standardized YLL, YLD, and DALY rates of AN and BN in 2019 stratified by sex are presented in Table 1. It was estimated that the ASIR [18.80 (95% UI: 13.14–25.94) vs. 8.26 (95% UI: 5.93–11.34) per 100,000 population], ASPR [57.43 (95% UI: 39.30–85.01) vs. 20.27 (95% UI: 13.97–29.11) per 100,000 population], and age-standardized DALY rate [12.60 (95% UI: 7.31–20.50) vs. 4.49 (95% UI: 2.58–7.06) per 100,000 population] of AN among females was significantly higher than that among males; however, men had a significantly higher ASIR of BN than women [194.31 (95% UI: 124.94–278.42) vs. 59.36 (95% UI: 37.92–87.51) per 100,000]. As shown in Table 2, the ASIR [13.22 (95% UI: 9.35–18.23) vs. 14.23 (95% UI: 10.11–19.54) per 100,000], ASPR [38.08 (95% UI: 26.37–55.73) vs. 42.78 (95% UI: 30.09–60.91) per 100,000], and age-standardized DALYs [8.38 (95% UI: 4.87–13.35) vs. 9.26 (95% UI: 5.45–14.65) per 100,000] of AN were lower in China than those in middle-SDI regions. Compared with middle-SDI regions, the ASIR [130.05 (95% UI: 84.02–187.13) vs. 136.42 (95% UI: 88.92–195.15) per 100,000], ASPR [75.21 (95% UI: 48.52–105.97) vs. 107.01 (95% UI: 71.53–147.92) per 100,000], and age-standardized DALYs [16.16 (95% UI: 9.23–25.40) vs. 22.70 (95% UI: 13.03–35.13) per 100,000] of BN in China were lower.

**Table 1:** Trends in the burden of anorexia nervosa and bulimia nervosa between males and females from 1990 to 2019.

**Variable**	**Males**			**Females**		
	**Rates in 1990 (95% UI)**	**Rates in 2019 (95% UI)**	**AAPC, % (95% CI)**	**Rates in 1990 (95% UI)**	**Rates in 2019 (95% UI)**	**AAPC, % (95% CI)**
**Age-standardized rate of anorexia nervosa, per 100,000 population**						
Prevalence	13.29 (9.19–19.15)	20.27 (13.97–29.11)	1.5 (1.4–1.5)	35.96 (24.72–52.27)	57.43 (39.3–85.01)	1.6 (1.6–1.7)
Incidence	5.74 (4.10–7.94)	8.26 (5.93–11.34)	1.3 (1.2–1.3)	12.61 (8.89–17.72)	18.8 (13.14–25.94)	1.4 (1.4–1.4)
YLLs	0.01 (0.00–0.01)	0.08 (0.06–0.13)	9.9 (8.9–10.9)	0.10 (0.06–0.16)	0.19 (0.14–0.25)	2.1 (1.2–3.0)
YLDs	2.89 (1.66–4.61)	4.41 (2.50–6.98)	1.5 (1.4–1.5)	7.71 (4.47–12.57)	12.40 (7.12–20.3)	1.7 (1.6–1.7)
DALYs	2.90 (1.66–4.61)	4.49 (2.58–7.06)	1.5 (1.5–1.6)	7.81 (4.57–12.64)	12.60 (7.31–20.5)	1.7 (1.6–1.7)
**Age-standardized rate of bulimia nervosa, per 100,000 population**						
Prevalence	40.76 (25.29–57.59)	77.15 (48.99–110.09)	2.2 (2.2–2.3)	43.94 (28.87–60.88)	73.15 (48.52–100.92)	1.8 (1.7–1.8)
Incidence	128.74 (82.73–184.84)	194.31 (124.94–278.42)	1.4 (1.4–1.5)	42.62 (26.99–63.18)	59.36 (37.92–87.51)	1.1 (1.1–1.2)
YLLs	0.00 (0.00–0.01)	0.03 (0.01–0.05)	7.8 (7.0–8.6)	0.01 (0.00–0.01)	0.02 (0.01–0.03)	4.4 (3.7–5.1)
YLDs	8.78 (4.88–13.99)	16.63 (9.28–26.25)	2.2 (2.2–2.3)	9.30 (5.39–14.7)	15.61 (8.89–24.73)	1.8 (1.8–1.9)
DALYs	8.79 (4.88–14.00)	16.67 (9.32–26.29)	2.2 (2.2–2.3)	9.31 (5.40–14.71)	15.63 (8.91–24.75)	1.8 (1.8–1.9)

*AAPC, average annual percentage change; DALYs, disability-adjusted life years; YLLs, years of life lost; YLDs, years lived with disability*.

**Table 2:** Comparison of age-standardized incidence, prevalence, YLLs, YLDs, and DALY rates of eating disorders between China and middle-sociodemographic index (SDI) regions in 2019.

**Variable**,	**Anorexia nervosa**		**Bulimia nervosa**	
**per 100,000 (95% UI) population**	**China**	**Middle-SDI regions**	**China**	**Middle-SDI regions**
Age-standardized incidence	13.22 (9.35–18.23)	14.23 (10.11–19.54)	130.05 (84.02–187.13)	136.42 (88.92–195.15)
Age-standardized prevalence	38.08 (26.37–55.73)	42.78 (30.09–60.91)	75.21 (48.52–105.97)	107.01 (71.53–147.92)
Age-standardized YLLs	0.14 (0.11–0.17)	0.09 (0.07–0.13)	0.03 (0.02–0.04)	0.02 (0.01–0.03)
Age-standardized YLDs	8.24 (4.73–13.20)	9.16 (5.37–14.56)	16.14 (9.20–25.37)	22.68 (13.01–35.12)
Age-standardized DALYs	8.38 (4.87–13.35)	9.26 (5.46–14.65)	16.16 (9.23–25.40)	22.7 (13.03–35.13)

*DALYs, disability-adjusted life years; YLLs, years of life lost; YLDs, years lived with disability*.

### Eating Disorder Burden Stratified by Age and Sex in 2019

**Figure 1:**
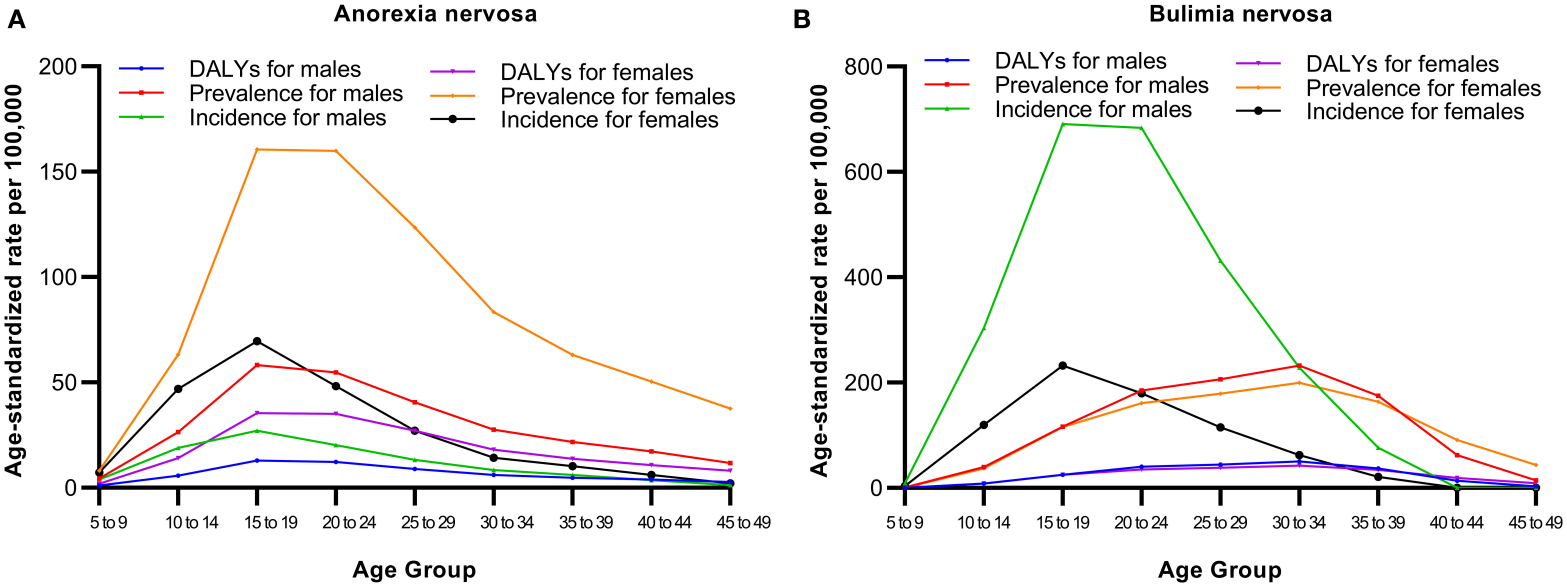
Prevalence, incidence, and DALY rates of anorexia nervosa **(A)** and bulimia nervosa **(B)** by age in China, 2019.

The highest prevalence, incidence, and DALY rates of AN were observed in teenagers aged 15–19 years (Figure 1). The prevalence, incidence, and DALY rates of AN increased gradually with age from 5 to 14 years, peaked at 15–19 years, and then decreased gradually with age from 20 to 49 years.

Similar to the trend of the incidence rate in AN, an inverse U-shaped trend of the incidence rate for BN with age was observed (Figure 1). The incidence rate of BN increased gradually with age from 5 to 14 years, peaked at 15–19 years, and then decreased gradually with age from 20 to 49 years. The figure shows that the age difference exists in the prevalence and DALY rates of BN compared with the incidence rate, which peaked at 30–34 years among males and females.

### Trends in Eating Disorder Burdens From 1990 to 2019

**Figure 2:**
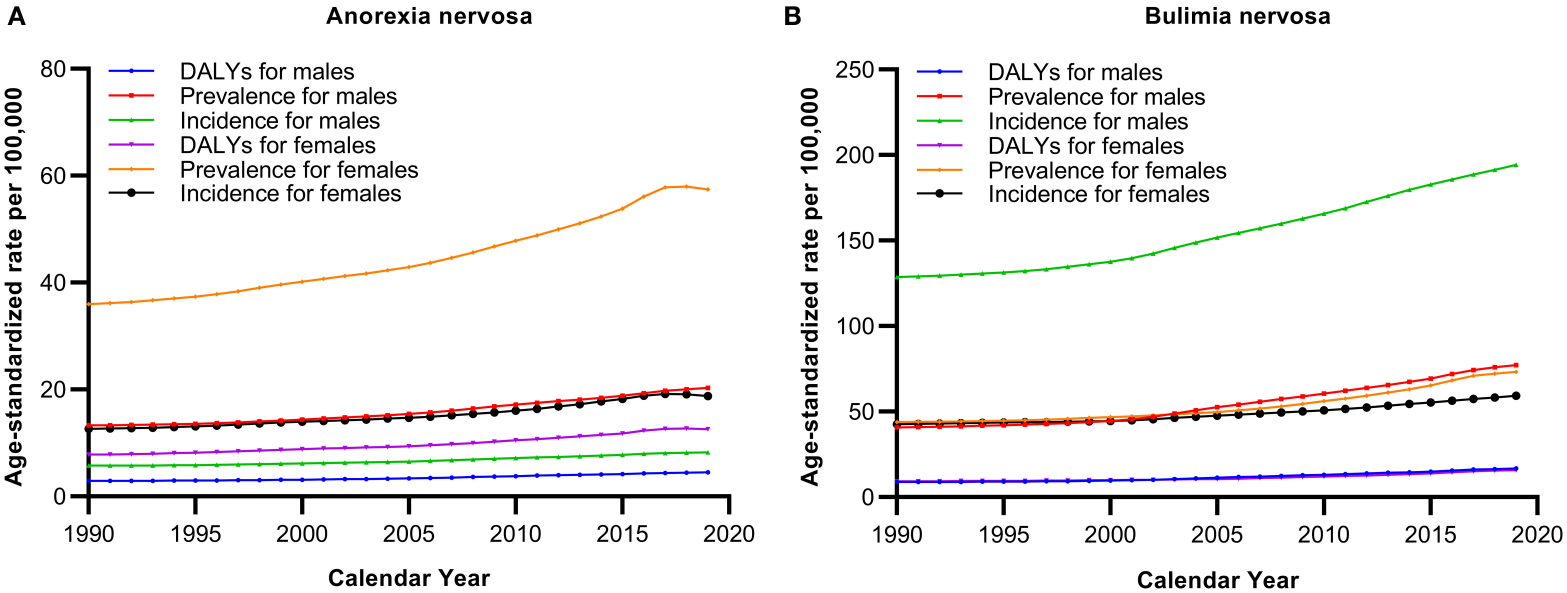
Trends in age-standardized prevalence, incidence, and DALY rates of anorexia nervosa **(A)** and bulimia nervosa **(B)** in China from 1990 to 2019.

As shown in Figure 2 and Table 3, the ASIRs, ASPRs and age-standardized DALYs of AN and BN gradually increased from 1990 to 2019 in China. The average APCs in the ASPR [2.0% (95% CI: 2.0–2.1%) vs. 1.6% (95% CI: 1.5–1.6%)] and age-standardized DALYs [2.0% (95% CI: 2.0–2.1%) vs. 1.6% (95% CI: 1.5–1.6%)] were significantly higher in BN than in AN. The periods with the fastest significant APCs in the ASIRs were 2012–2017 for AN [2.4% (95% CI: 2.3–2.5%)] and 2010–2015 for BN [2.1% (95% CI: 2.1–2.1%)]. In addition, the periods with the fastest significant APCs in the ASPR [3.0% (95% CI: 2.6–3.5%) for AN; 3.7% (95% CI: 3.5–4.0%) for BN] and age-standardized DALY rates [3.0% (95% CI: 2.4–3.7%) for AN; 3.7% (95% CI: 3.4–4.0%) for BN] were 2014–2017 for both AN and BN.

**Table 3:** Annual percentage changes in the eating disorder burden from 1990 to 2019 in China by joinpoint regression analysis.

**Variable**	**AAPC**	**Trend 1**		**Trend 2**		**Trend 3**		**Trend 4**		**Trend 5**		**Trend 6**	
	**1990-2019**	**Year**	**APC**	**Year**	**APC**	**Year**	**APC**	**Year**	**APC**	**Year**	**APC**	**Year**	**APC**
**Age-standardized rate of anorexia nervosa**													
Prevalence	1.6 (1.5–1.6)	1990–1995	0.7 (0.6–0.8)	1995–2005	1.4 (1.4–1.5)	2005–2014	2.1 (2.1–2.2)	2014–2017	3.0 (2.6–3.5)	2017–2019	0.0 (−0.4–0.5)		
Incidence	1.3 (1.3–1.4)	1990–1995	0.6 (0.5–0.7)	1995–2000	1.2 (1.1–1.4)	2000–2006	1.1 (1.0–1.2)	2006–2012	1.9 (1.8–2.0)	2012–2017	2.4 (2.3–2.5)	2017–2019	−0.5 (−0.9–−0.1)
YLLs	3.7 (2.1–5.3)	1990–1999	6.8 (5.4–8.1)	1999–2006	−2.4 (−4.6–−0.1)	2006–2009	14.7 (−0.1–31.6)	2009–2019	2.2 (1.1–3.3)				
YLDs	1.6 (1.5–1.7)	1990–1995	0.7 (0.6–0.8)	1995–2005	1.5 (1.4–1.5)	2005–2014	2.1 (2.1–2.2)	2014–2017	3.0 (2.4–3.5)	2017–2019	0.1 (−0.5–0.6)		
DALYs	1.6 (1.5–1.7)	1990–1994	0.6 (0.4–0.8)	1994–2006	1.4 (1.4–1.5)	2006–2010	2.5 (2.2–2.8)	2010–2014	2.0 (1.7–2.3)	2014–2017	3.0 (2.4–3.7)	2017–2019	0.1 (−0.5–0.7)
**Age-standardized rate of bulimia nervosa**													
Prevalence	2.0 (2.0–2.1)	1990–1996	0.5 (0.5–0.5)	1996–2001	1.2 (1.1–1.3)	2001–2007	2.6 (2.5–2.6)	2007–2014	2.8 (2.7–2.8)	2014–2017	3.7 (3.5–4)	2017–2019	1.8 (1.5–2.1)
Incidence	1.4 (1.4–1.4)	1990–1997	0.5 (0.5–0.5)	1997-2001	0.9 (0.9–1)	2001–2004	2 (1.9–2.1)	2004–2010	1.7 (1.7–1.7)	2010–2015	2.1 (2.1–2.1)	2015–2019	1.7 (1.7–1.7)
YLLs	6.3 (5.5–7.1)	1990–2000	−0.1 (−0.5–0.4)	2000–2004	5.2 (2.2–8.4)	2004–2007	21.8 (14.9–29.2)	2007–2012	13 (10.9–15.1)	2012–2019	5.4 (4.6–6.2)		
YLDs	2.0 (2.0–2.1)	1990–1996	0.5 (0.5 to 0.6)	1996–2001	1.2 (1.2–1.3)	2001–2007	2.6 (2.5–2.7)	2007–2014	2.8 (2.7–2.8)	2014–2017	3.7 (3.4–4)	2017–2019	1.8 (1.6–2.1)
DALYs	2.0 (2.0–2.1)	1990–1996	0.5 (0.5 to 0.6)	1996–2001	1.2 (1.1–1.3)	2001–2007	2.6 (2.5–2.7)	2007–2014	2.8 (2.7–2.8)	2014–2017	3.7 (3.4–4)	2017–2019	1.8 (1.6–2.1)

*AAPC, average annual percentage change; DALYs, disability-adjusted life years; YLLs, years of life lost; YLDs, years lived with disability*.

**Figure 3:**
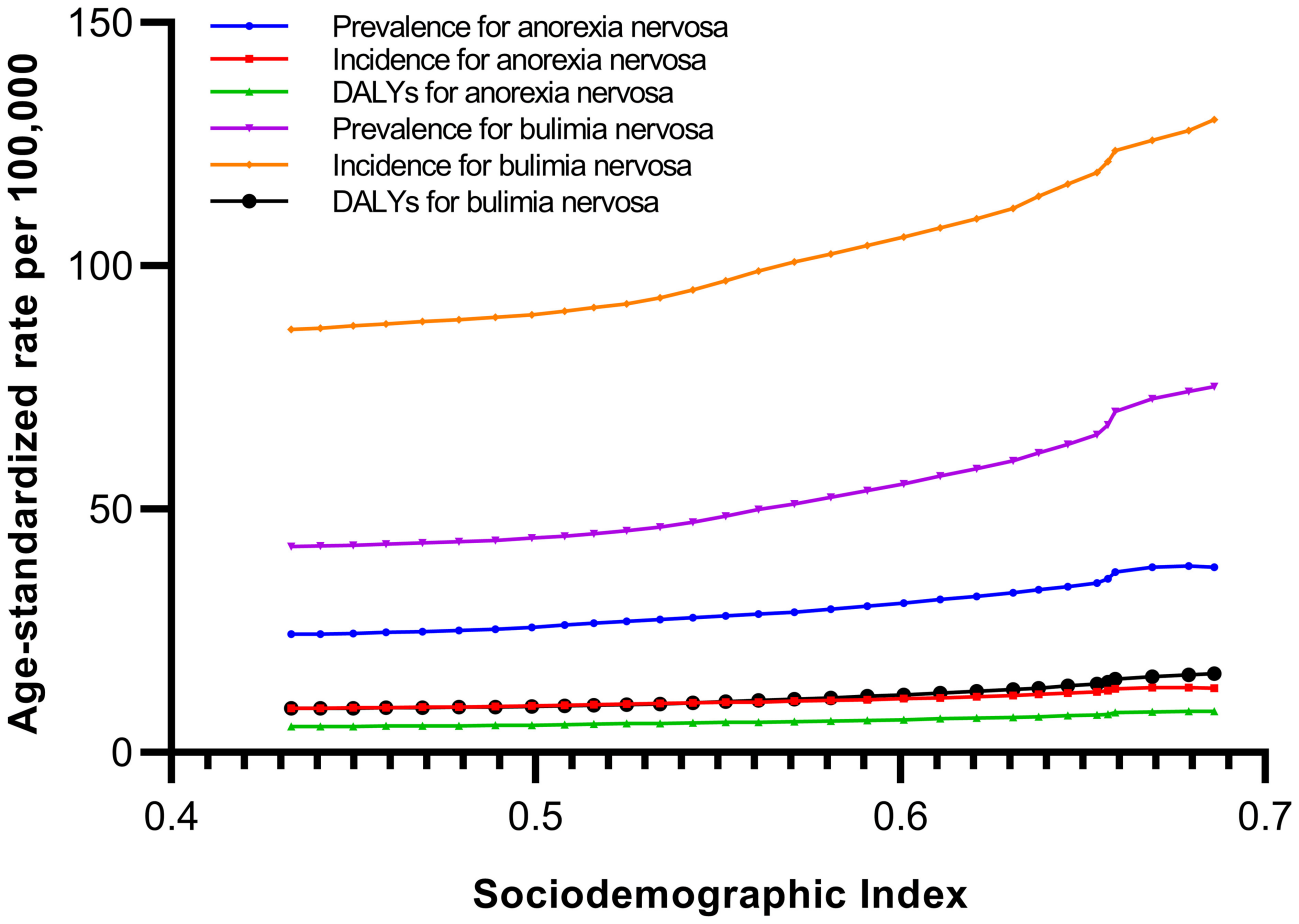
Age-standardized prevalence, incidence, and DALY rates of anorexia nervosa and bulimia nervosa in China by sociodemographic index, 1990–2019.

Figure 3 presents the observed ASIR, ASPR, and age-standardized DALY rates from 1990 to 2019 and their positive associations with the SDI in China. The expected trend of burden estimates was linear in nature, increasing with increasing SDI values. Moreover, the ASIR and ASPR of BN tended to increase more rapidly with increasing SDI values from 1990 to 2019 in China.

## Discussion

To our knowledge, the present study was the first comprehensive evaluation of the considerable and ever-increasing trends in prevalence and DALYs of AN and BN in China over the past three decades based on GBD 2019 data. The standardized methods for estimating eating disorder metrics used in the GBD 2019 made it possible to compare regional-level metrics with those in China. We examined the disease burden of eating disorders in China. In 2019, there were 112.62 (95% CI, 83.53–145.30) patients per 100,000 population suffering from AN and BN in China. According to the latest national survey in 2012, anxiety disorders were the most common class of mental disorders (7.6%), followed by mood disorders (7.4%), substance-use disorders (4.7%), impulse-control disorders (1.5%), and eating disorders (0.1%) in China (32). Although the prevalence is relatively low compared with other mental disorders, given the massive population base of China, it was estimated that more than 1.5 million people were affected by this disease in 2019 (32, 33).

The patterns of AN and BN burdens varied by age and sex in our study. Similar to most mental disorders globally, there was an inverse U-shaped trend for the prevalence, incidence and DALYs of AN and BN with age. The highest incidence rates of AN and BN were observed in the 15–19 year age group, which was consistent with previous studies that have suggested AN and BN to be most newly diagnosed in adolescents (14). However, the prevalence and DALY rates of BN reached a peak at the age of 30–34 years. This finding is consistent with previous results (19). This may be due to the insufficient treatment of adolescent BN patients by clinical psychiatrists and the poor treatment effect. Consequently, in the following 10 years, the disease continued to affect that population. Therefore, public health policy should pay more attention to clinicians, especially primary care physicians, to improve the capability of diagnosis and treatment of the disease to promote the reduction of the disease burden of BN.

A female predominance in the age-standardized rates was observed for AN, whereas males had a higher burden of BN than women. Two longitudinal studies have shown that the proportion of men with eating disorders is much lower than that of women, accounting for only 5–12% of the total population with eating disorders (34, 35). A study from Hong Kong indicated that the clinical awareness of male BN was very low from 1987 to 2007 (13). However, with the development of the economy, the improvement of living standards, and the deepening of social esthetic influence, recent studies have reported that the proportion of male patients has increased in Spain and Australia (36–38). The majority of male patients do not realize their symptoms in time or are misdiagnosed by doctors as having other diseases, resulting in a low detection rate of eating disorders in male patients. The higher incidence of male patients with BN might merely reflect under-recognition in previous decades. In addition, BN's increasing trend may be attributed to the improved awareness of mental disorders and changing diagnostic practices among male patients. Compared with a little knowledge of eating disorders in the male population, it is important to widespread the comprehension taking into account the gender perspective. Attention must be paid particularly to critical developmental periods, such as puberty and adolescence, where early detection and prevention of these disorders is of greatest importance, inside a conceptual framework that considers the mutual influence between biological and psychological vulnerability and sociocultural factors. Obtaining an accurate diagnosis and effective treatment has become the primary reason for the lack of appropriate treatment for many potential eating disorder patients. Therefore, positive concern and support should be provided to male patients.

The ASPR, ASIR and age-standardized DALYs of AN and BN in China were lower than those in the middle-SDI regions in 2019. This could be partly explained by differences in economic growth and sociohistorical factors in China (21). However, given the very limited information on this issue, further studies in China are needed to explore the potential reason. The aforementioned study found that the burden of eating disorders increased with the increased SDI at regional levels and the highest burden was observed in the high SDI regions, particularly in Australasia, Western Europe, and high-income North America (19). In general, overweight and obesity are positively correlated with economic levels, globalization, and urbanization; these conditions could increase the risk of eating disorders (39). In the past two decades, China's society and economy have developed rapidly, and urbanization has continued to spread (40). The use of media, especially the Internet, has become popular; it provides people with rich information that guides their behavioral choices (41). However, multiculturalism has introduced people to the Western concept of “slimness is beauty.” As a result, women, especially adolescents, take improper weight loss measures and change their eating habits to achieve this goal, thereby increasing the risk of eating disorders (42). Data from the China Health and Nutrition Survey in 2009 showed that the detection rate of eating disorders among young females (18–35 years old) was 6.3%, and female teenagers (12–17 years old) had a detection rate of 7.8%. Compared with Western countries (13.5–26.9%), the detection rate is still low in China (43). However, the obesity rate in Chinese females has shown a significant increasing trend over the past two decades (44, 45). Concepts of weight loss and eating habits as well as overweight or obesity are related to eating disorders and are widely spread through the media (46).

This study analyzes the current conditions and changes in prevalence, incidence, and DALYs of eating disorders and the association with annual SDI over the past three decades in China. Our research reveals a potential increasing burden of BN among males. However, the interpretation of our study has several limitations. First, binge eating disorders, as some of the most common eating disorders, were not included in GBD 2019. Therefore, conclusions about eating disorders in general should be made with caution. Second, there is considerable heterogeneity in terms of data density for each region location-cause combination in the GBD study. Given that the GBD data are based on estimated prevalence based on global average and country-level covariates for geographies with no data, it follows that the epidemiology data and burden of disease estimates may not be accurate. More studies of epidemiology and risk factors at the national level are needed to reduce its bias (26). Third, changes in socioeconomic structures and diagnostic criteria should also be considered when evaluating the change trend of the eating disorder burden in China.

## Conclusions

From 1990 to 2019, the burden of eating disorders increased in China. A higher burden of BN was observed in males than in females, and a higher prevalence and DALYs of BN were observed in young individuals aged 30–34 than in individuals in other age groups. An enhanced understanding of disparities in disease burden could facilitate the early identification of individuals at risk of developing eating disorders, thereby helping to take timely interventions that effectively reduce the burden of eating disorders in China.

## Data Availability Statement

The datasets generated for this study can be found in the GBD at http://ghdx.healthdata.org/gbd-results-tool.

## Author Contributions

ZL and YL conceived and designed the study and drafted the manuscript. HG and CH supervised the study. PC did the statistical analysis. ZL, LW, HG, CH, and AL contributed to acquisition, analysis, or interpretation of data. All authors revised the report and approved the final version before submission.

## Conflict of Interest

The authors declare that the research was conducted in the absence of any commercial or financial relationships that could be construed as a potential conflict of interest.

## References

[1] Mitchell JE Peterson CB. Anorexia nervosa. N Engl J Med. (2020) 382:1343–51. 10.1056/NEJMcp1803175 32242359

[2] Wade TD. Recent research on bulimia Nervosa. Psychiatr Clin North Am. (2019) 42:21–32. 10.1016/j.psc.2018.10.002 30704637

[3] Treasure J Duarte TA Schmidt U. Eating disorders. Lancet. (2020) 395:899–911. 10.1016/S0140-6736(20)30059-3 32171414

[4] Duncan L Yilmaz Z Gaspar H Walters R Goldstein J Anttila V. Significant locus and metabolic genetic correlations revealed in genome-wide association study of Anorexia Nervosa. Am J Psychiatr. (2017) 174:850–8. 10.1176/appi.ajp.2017.16121402 28494655 PMC5581217

[5] Watson HJ Yilmaz Z Thornton LM. Genome-wide association study identifies eight risk loci and implicates metabo-psychiatric origins for anorexia nervosa. Nat Genet. (2019) 51:1207–14. 10.1038/s41588-019-0439-2 31308545 PMC6779477

[6] Su X Liang H Yuan W Olsen J Cnattingius S Li J. Prenatal and early life stress and risk of eating disorders in adolescent girls and young women. Eur Child Adolesc Psychiatr. (2016) 25:1245–53. 10.1007/s00787-016-0848-z 27083434 PMC5083758

[7] Larsen JT Munk-Olsen T Bulik CM Thornton LM Koch SV Mortensen PB. Early childhood adversities and risk of eating disorders in women: a Danish register-based cohort study. Int J Eat Disord. (2017) 50:1404–12. 10.1002/eat.22798 29105808

[8] Arcelus J Mitchell AJ Wales J Nielsen S. Mortality rates in patients with anorexia nervosa and other eating disorders. A meta-analysis of 36 studies. Arch Gen Psychiatr. (2011) 68:724–31. 10.1001/archgenpsychiatry.2011.74 21727255

[9] Johnson JG Cohen P Kasen S Brook JS. Eating disorders during adolescence and the risk for physical and mental disorders during early adulthood. Arch Gen Psychiatr. (2002) 59:545–52. 10.1001/archpsyc.59.6.545 12044197

[10] Padierna A Martín J Aguirre U González N Muñoz P Quintana JM. Burden of caregiving amongst family caregivers of patients with eating disorders. Soc Psychiatr Psychiatr Epidemiol. (2013) 48:151–61. 10.1007/s00127-012-0525-6 22722535

[11] Preti A Rocchi MB Sisti D Camboni MV Miotto P. A comprehensive meta-analysis of the risk of suicide in eating disorders. Acta Psychiatr Scand. (2011) 124:6–17. 10.1111/j.1600-0447.2010.01641.x 21092024

[12] Swinbourne J Hunt C Abbott M Russell J St Clare T Touyz S. The comorbidity between eating disorders and anxiety disorders: prevalence in an eating disorder sample and anxiety disorder sample. Aust N Z J Psychiatr. (2012) 46:118–31. 10.1177/0004867411432071 22311528

[13] Wang LY Nichols LP Austin SB. The economic effect of Planet Health on preventing bulimia nervosa. Arch Pediatr Adolesc Med. (2011) 165:756–62. 10.1001/archpediatrics.2011.105 21810638

[14] Erskine HE Whiteford HA Pike KM. The global burden of eating disorders. Curr Opin Psychiatr. (2016) 29:346–53. 10.1097/YCO.0000000000000276 27532942

[15] Whiteford HA Degenhardt L Rehm J Baxter AJ Ferrari AJ Erskine HE. Global burden of disease attributable to mental and substance use disorders: findings from the Global Burden of Disease Study 2010. Lancet. (2013) 382:1575–86. 10.1016/S0140-6736(13)61611-6 23993280

[16] Currin L Schmidt U Treasure J Jick H. Time trends in eating disorder incidence. Br J Psychiatr. (2005) 186:132–5. 10.1192/bjp.186.2.132 15684236

[17] Milos G Spindler A Schnyder U Martz J Hoek HW Willi J. Incidence of severe anorexia nervosa in Switzerland: 40 years of development. Int J Eat Disord. (2004) 35:250–8. 10.1002/eat.10244 15048940

[18] van Son GE van Hoeken D Bartelds AI van Furth EF Hoek HW. Time trends in the incidence of eating disorders: a primary care study in the Netherlands. Int J Eat Disord. (2006) 39:565–9. 10.1002/eat.20316 16791852

[19] Wu J Liu J Li S Ma H Wang Y. Trends in the prevalence and disability-adjusted life years of eating disorders from 1990 to 2017: results from the Global Burden of Disease Study 2017. Epidemiol Psychiatr Sci. (2020) 29:e191. 10.1017/S2045796020001055 33283690 PMC7737181

[20] Lee S Chan YY Hsu LK. The intermediate-term outcome of Chinese patients with anorexia nervosa in Hong Kong. Am J Psychiatr. (2003) 160:967–72. 10.1176/appi.ajp.160.5.967 12727702

[21] Lee S Ng KL Kwok K Fung C. The changing profile of eating disorders at a tertiary psychiatric clinic in Hong Kong (1987-2007). Int J Eat Disord. (2010) 43:307–14. 10.1002/eat.20686 19350649

[22] Huon GF Mingyi Q Oliver K Xiao G. A large-scale survey of eating disorder symptomatology among female adolescents in the People's Republic of China. Int J Eat Disord. (2002) 32:192–205. 10.1002/eat.10061 12210662

[23] Jackson T Chen H. Predicting changes in eating disorder symptoms among Chinese adolescents: a 9-month prospective study. J Psychosom Res. (2008) 64:87–95. 10.1016/j.jpsychores.2007.08.015 18158004

[24] Tong J Miao S Wang J Yang F Lai H Zhang C. A two-stage epidemiologic study on prevalence of eating disorders in female university students in Wuhan, China. Soc Psychiatry Psychiatr Epidemiol. (2014) 49:499–505. 10.1007/s00127-013-0694-y 23744441

[25] Wu J Lin Z Liu Z He H Bai L Lyu J. Secular trends in the incidence of eating disorders in China from 1990 to 2017: a joinpoint and age-period-cohort analysis. Psychol Med. (2020) 1–11. 10.1017/S0033291720002706 32744194

[26] GBD 2019 Diseases and Injuries Collaborators. Global burden of 369 diseases and injuries in 204 countries and territories, 1990-2019: a systematic analysis for the Global Burden of Disease Study 2019. Lancet. (2020) 396:1204–22. 10.1016/S0140-6736(20)30925-9 33069326 PMC7567026

[27] American Psychiatric Association. Diagnostic and Statistical Manual of Mental Disorders, 4th ed. Washington: American Psychiatric Association (1994).

[28] American Psychiatric Association. Desk Reference to the Diagnostic Criteria From DSM-5. Arlington: American Psychiatric Association (2013).

[29] World Health Organization. The International Classification of Diseases (10th revison). Geneva: World Health Organization (1992).

[30] Kim HJ Fay MP Feuer EJ Midthune DN. Permutation tests for joinpoint regression with applications to cancer rates. Stat Med. (2000) 19:335–51. 10.1002/(SICI)1097-0258(20000215)19:3&lt;335::AID-SIM336&gt;3.0.CO;2-Z 10649300

[31] Clegg LX Hankey BF Tiwari R Feuer EJ Edwards BK. Estimating average annual per cent change in trend analysis. Stat Med. (2009) 28:3670–82. 10.1002/sim.3733 19856324 PMC2843083

[32] Huang Y Wang Y Wang H Liu Z Yu X Yan J. Prevalence of mental disorders in China: a cross-sectional epidemiological study. Lancet Psychiatr. (2019) 6:211–24. 10.1016/S2215-0366(18)30511-X 30792114

[33] National Bureau of Statistics of China. Tabulation on population census of the People's Republic of China. (2020). Available online at: http://www.stats.gov.cn/tjsj/pcsj.

[34] Button E Aldridge S Palmer R. Males assessed by a specialized adult eating disorders service: patterns over time and comparisons with females. Int J Eat Disord. (2008) 41:758–61. 10.1002/eat.20553 18528870

[35] Haynos AF Wall MM Chen C Wang SB Loth K Neumark-Sztainer D. Patterns of weight control behavior persisting beyond young adulthood: results from a 15-year longitudinal study. Int J Eat Disord. (2018) 51:1090–7. 10.1002/eat.22963 30353938 PMC6391054

[36] Fernández-Aranda F Murcia S. Evidence-Guided Treatment for Males With Eating Disorders (2014).

[37] Rodríguez-Cano T Beato-Fernández L Belmonte-Llario A. New contributions to the prevalence of eating disorders in Spanish adolescents: detection of false negatives. Eur Psychiatr. (2005) 20:173–8. 10.1016/j.eurpsy.2004.04.002 15797703

[38] Mitchison D Hay P Slewa-Younan S Mond J. The changing demographic profile of eating disorder behaviors in the community. BMC Public Health. (2014) 14:943. 10.1186/1471-2458-14-943 25213544 PMC4246495

[39] Dong Y Jan C Ma Y Dong B Zou Z Yang Y. Economic development and the nutritional status of Chinese school-aged children and adolescents from 1995 to 2014: an analysis of five successive national surveys. Lancet Diabetes Endocrinol. (2019) 7:288–99. 10.1016/S2213-8587(19)30075-0 30902266

[40] Yang XJ. China's rapid urbanization. Science. (2013) 342:310. 10.1126/science.342.6156.310-a 24136949

[41] Zhou R Fong PS Tan P. Internet use and its impact on engagement in leisure activities in China. PLoS ONE. (2014) 9:e89598. 10.1371/journal.pone.0089598 24586902 PMC3931801

[42] Walker M Thornton L De Choudhury M Teevan J Bulik CM Levinson CA. Facebook use and disordered eating in college-aged women. J Adolesc Health. (2015) 57:157–63. 10.1016/j.jadohealth.2015.04.026 26206436 PMC4514918

[43] Watson HJ Hamer RM Thornton LM Peat CM Kleiman SC Du S. Prevalence of screening-detected eating disorders in chinese females and exploratory associations with dietary practices. Eur Eat Disord Rev. (2015) 23:68–76. 10.1002/erv.2334 25407415 PMC4314345

[44] Li Y Teng D Shi X Qin G Qin Y Quan H. Prevalence of diabetes recorded in mainland China using 2018 diagnostic criteria from the American Diabetes Association: national cross sectional study. BMJ. (2020) 369:m997. 10.1136/bmj.m997 32345662 PMC7186854

[45] Mi YJ Zhang B Wang HJ Yan J Han W Zhao J. Prevalence and secular trends in obesity among chinese adults, 1991-2011. Am J Prev Med. (2015) 49:661–9. 10.1016/j.amepre.2015.05.005 26275960 PMC4615397

[46] Musaiger AO Al-Mannai M Tayyem R Al-Lalla O Ali EYA Kalam F. Risk of disordered eating attitudes among adolescents in seven Arab countries by gender and obesity: a cross-cultural study. Appetite. (2013) 60:162–7. 10.1016/j.appet.2012.10.012 23092757

